# Molecular Regulations and Functions of the Transient Receptor Potential Channels of the Islets of Langerhans and Insulinoma Cells

**DOI:** 10.3390/cells9030685

**Published:** 2020-03-11

**Authors:** Md. Shahidul Islam

**Affiliations:** 1 Karolinska Institutet, Department of Clinical Science and Education, Södersjukhuset, Research Center, 5th floor, SE-118 83 Stockholm, Sweden; shahidul.islam@ki.se; 2 Department of Emergency Care and Internal Medicine, Uppsala University Hospital, Uppsala University, SE-751 85 Uppsala, Sweden

**Keywords:** TRP channels of islets, TRP channels and insulin, TRPC1, TRPM2, TRPM3, TRPM4, TRPM5, TRPM7, TRPA1, TRP channels and GLP-1

## Abstract

Insulin secretion from the β-cells of the islets of Langerhans is triggered mainly by nutrients such as glucose, and incretin hormones such as glucagon-like peptide-1 (GLP-1). The mechanisms of the stimulus-secretion coupling involve the participation of the key enzymes that metabolize the nutrients, and numerous ion channels that mediate the electrical activity. Several members of the transient receptor potential (TRP) channels participate in the processes that mediate the electrical activities and Ca^2+^ oscillations in these cells. Human β-cells express TRPC1, TRPM2, TRPM3, TRPM4, TRPM7, TRPP1, TRPML1, and TRPML3 channels. Some of these channels have been reported to mediate background depolarizing currents, store-operated Ca^2+^ entry (SOCE), electrical activity, Ca^2+^ oscillations, gene transcription, cell-death, and insulin secretion in response to stimulation by glucose and GLP1. Different channels of the TRP family are regulated by one or more of the following mechanisms: activation of G protein-coupled receptors, the filling state of the endoplasmic reticulum Ca^2+^ store, heat, oxidative stress, or some second messengers. This review briefly compiles our current knowledge about the molecular mechanisms of regulations, and functions of the TRP channels in the β-cells, the α-cells, and some insulinoma cell lines.

## 1. Introduction

The islets of Langerhans contain mainly the insulin-secreting β-cells, glucagon-secreting α-cells, and somatostatin-secreting δ-cells [1]. Because of the difficulty in obtaining pure human β-cells, it is common to use a variety of rodent insulinoma cells and glucagonoma cells for basic research in this field. The β-cells secrete insulin in response to stimulation by nutrients such as glucose, amino acids, and free fatty acids, neurotransmitters such as acetylcholine, and incretin hormones such as glucagon-like peptide-1 (GLP-1) [2]. The molecular mechanisms of stimulus-secretion coupling in the β-cells involve the intermediary metabolism of the nutrients in the cytoplasm and in the mitochondria, the participation of some G protein-coupled receptors (GPCR), and many ion channels [2]. Crucial events in the stimulus-secretion coupling are electrical activities, and increase in the concentration of Ca^2+^ in the cytoplasm ([Ca^2+^]_i_), in the form of spikes, bursts, and oscillations [3]. The electrical activities and the [Ca^2+^]_i_ oscillations are generated by concerted participation of a unique repertoire of ion channels present in the β-cells [4]. These include different K^+^ channels, Ca^2+^ channels, Na^+^ channels, Cl^−^ channels, volume-sensitive anion channels, hyperpolarization-activated cyclic nucleotide-gated channels, store-operated Ca^2+^ entry (SOCE) channels, and the transient receptor potential (TRP) channels [4]. It is not meaningful to debate which of these ion channels are more important than the others. Study of the ion channels of the islets is important because of their roles in the secretion of the hormones and in the impairment of such secretions in the pathogenesis of diabetes mellitus, which is a major public health problem.

Many studies have demonstrated the presence of different TRP channels in the islets, different insulinoma cells, and glucagonoma cells by different methods including functional studies using pharmacological tools, RT-PCR, RNA-sequencing, Western blot, immuno-histochemistry, immuno-fluorescence, and electrophysiology. Interpretation of some of the results, especially those obtained by antibody-based methods may be difficult. Demonstration of expression of TRP channels at RNA or protein level does not necessarily mean that they are translocated to the plasma membrane and form functional channels. Interpretation of the mRNA expression data can also be difficult. For instance, mRNA level may be low while the protein level may still be high because of high RNA degradation rate, but slow protein turnover rate. Similarly, mRNA level may be high, but the protein level may be very low due to repression of translation. Moreover, the methods used for purification of the e.g., fluorescence activated cell sorting (FACS) may alter the mRNA expression level. Some TRP channels are expressed in rodent islets and rodent insulinoma or glucagonoma cell lines but are almost absent in human islets.

During recent years, interest in the understanding the roles of the TRP channels in the physiology and pathology of islets in the context of diabetes has increased [5]. The availability of newer pharmacological tools and knockout mouse models have enabled the investigators elucidate the regulations and the functions of these channels in the islets. Today, our knowledge about these channels in the islets is substantially more than that about a decade back [6]. This review describes the essential background information, and the recent advances in our understanding of the regulation of these channels, and their roles in mediating β-cell functions.

## 2. TRPC1

**Figure 1:**
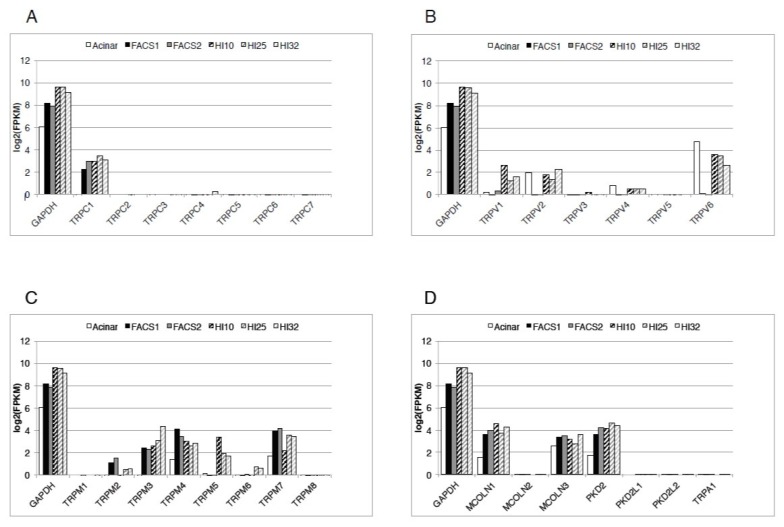
Expression of the transient receptor potential (TRP) channels in human β-cell and human islets. Expression levels are shown as bar plots on a log_2_(FPKM) scale. The bars in each group represent (from left to right) human pancreatic acinar cells; purified human β-cells preparations 1 and 2 (FACS1, FACS2); and human islet preparations HI10, HI25, and HI32. Relative levels of expressions of the channels of the TRPC family (**A**), TRPV family (**B**), TRPM family (**C**), and the remaining TRP channels (TRPA1, members of the TRPP, and TRPML families) (**D**), are shown. GAPDH expression is shown for comparison. A log_2_(FPKM) = 0 was considered as a minimum threshold for expression. MCOLN1 = TRPML1, MCOLN2 = TRPML2, MCOLN3 = TRPML3, PKD2 = TRPP1, PKD2L1 = TRPP2, PKD2L2 = TRPP3. FPKM = Fragments Per Kilobase Million. Reproduced with permission from [7], Marabita and Islam, 2017.

Among the channels of the TRPC family, only TRPC1 can be detected at mRNA level in human β-cells (Figure 1) [7]. TRPC1 mRNA can be detected in mouse islets [8,9], MIN6 cells [8,9], rat islets [10], rat beta cells [11], and INS-1 cells [10,11,12]. Rat primary β-cells express more TRPC1 compared to the INS-1 cells [11]. In MIN6 cells and mouse islets, four splice variants of *TRPC1* have been identified, the β-variant being the most abundant one [9]. Protein kinase C (PKC) is important for glucose-stimulated insulin secretion. In INS-1E cells, glucose increases insulin secretion by stimulation of PKCα, which induces phosphorylation of TRPC1 [12]. 

SOCE plays an important role in mediating insulin secretion [13]. In rat β-cells, TRPC1 and Orai1 form the non-selective cation channel that mediates SOCE and is regulated by STIM1 [10]. Orai1-mediated Ca^2+^ entry stimulates recruitment of TRPC1 into the plasma membrane. Orai1 and STIM1 form channels that are gated by STIM1 [14]. STIM1 gates TRPC1 by intermolecular electrostatic interaction between the positively charged poly-lysine domain in the C-terminus of STIM1 with the negatively charged aspartates in the TRPC1 [15]. SOCE is impaired in the β-cells obtained from patients with type 2 diabetes (T2D) [16]. The human *TRPC1* gene is located on the chromosome 3q23;the band 3q is associated with T2D [17,18]. Genetic polymorphisms of TRPC1 are associated with T2D and its complications in some populations [19]. In the Han Chinese population, the SNP rs7638459 has been suspected as a risk factor for T2D without diabetic nephropathy. The CC genotype of rs7638459 significantly increases risk compared with the TT genotype. In the same population another SNP, rs953239, is protective against development of nephropathy in T2D [19]. The CC genotype of rs953239 significantly reduces the risk of getting T2D without nephropathy compared to the AA genotype [19].

## 3. TRPC2, TRPC3, TRPC4, TRPC5 and TRPC6

In humans, *TRPC2* is a pseudogene and the protein is not expressed in human cells. TRPC2 is present in mouse insulinoma MIN6 cells [8].

TRPC3 is expressed in mouse and rat β-cells where it is triggered upon activation of some GPCRs. Activation of the G protein-coupled receptor 40 (GPR40) of rat β-cells by fatty acids potentiates glucose-induced insulin secretion. Activation of the GPR40 activates the TRPC3; this is mediated by activation of phospholipase C and the PKC pathway [20]. Activation of the TRPC3 channel induces a non-selective cation current that leads to depolarization of the membrane potential of the β-cell [20]. TRPC3 also plays a role in the development and proliferation of β-cells. The transcription factor pancreatic and duodenal homeobox 1 (Pdx-1) increases proliferation of islet cells partly by upregulating the expression of TRPC3 and TRPC6, and also by increasing the activity of these channels [21].

TRPC4 is not expressed in human β-cells (Figure 1) [7] but is expressed in rat and mouse primary β-cells and insulinoma cells (Table 1). At least two major isoforms of TRPC4 are known. TRPC4β lacks 84 amino acids in the C-terminus and TRPC4α is the full-length form. In INS-1 cells, TRPC4α is the main isoform, whereas in rat β-cells TRPC4β is the main isoform [11]. In βTC3 cells, TRPC4 is activated by store depletion, and this activates a non-selective cation current, which contributes to the generation of glucose-stimulated oscillations of membrane potential and [Ca^2+^]_i_ [8].

Leptin signaling activates the TRPC4 channel of β-cells through phosphorylation of the channel by phosphoinositide 3-kinase [22]. Reversible histidine phosphorylation plays an important role in the activation of the TRPC4 channel of β-cells. In INS-1 cells, protein histidine phosphatase 1 (PHPT-1) activates TRPC4 by dephosphorylating a histidine residue in the C-terminus of the channel [23]. Activation of TRPC4 increases [Ca^2+^]_i_, which leads to the translocation of the ATP-sensitive K^+^ (K_ATP_) channels to the plasma membrane. In *Phpt-1^−/−^* mice, TRPC4 is inhibited and the K_ATP_ current in the plasma membrane is decreased. Consistent with these observations, the *Phpt-1^−/−^* mice are hypoglycemic during the perinatal period [23].

Small GTPase Rasd1 activates TRPC4β, but not TRPC4α. Glucocorticoids increase Rasd1 in INS-1 cells, and by that way increase the TRPC4 current in these cells [24].

## 4. TRPM2

Early studies reported a non-selective Ca^2+^-permeable cation channel activated by β-NAD^+^, H_2_O_2_, and alloxan, and inhibited by AMP, in CRI-G1 rat insulinoma cells [25,26]. The current described has characteristics of TRPM2, e.g., unusually long single channel open times, linear current-voltage relationship, and the requirement of cytoplasmic Ca^2+^ for activation of the current [26,27]. Later on, it was demonstrated that the CRI-G1 cells express TRPM2 at a high level and adenosine 5′-diphosphate ribose (ADPR) activates the characteristic TRPM2 current [28]. Other rodent insulinoma cells that express TRPM2 are the INS-1E cells [29], the RIN-5F cells [30,31,32], and theHIT-T15 cells [33]. Primary mouse [31,34], rat [31], and human β-cells [7,29,35] express the TRPM2 channel. The α-cells do not express TRPM2 [31]. Human islets express two isoforms of the TRPM2 channel: the full length or the long form of the channel (TRPM2-L) and a short form (TRPM2-S) where the four C-terminal transmembrane domains, the pore region, and the entire C-terminus are truncated [29]. TRPM2-S, which does not form a channel, acts as a dominant negative of TRPM2-L [36]. The relative proportion of the two isoforms may determine the extent of the TRPM2-mediated Ca^2+^ influx.

ADPR together with the co-agonist Ca^2+^ activates TRPM2 [37]. The NUDT9 homology (NUDT9-H) domain of human TRPM2 plays crucial roles in mediating expression of the channel in the plasma membrane and in channel gating. ADPR binds to both the NUDT9-H domain and the TRPM homology regions (MHR) 1 and 2 (MHR1/2) [38]. The NUDT9-H domain of human TRPM2 binds ADPR and promotes channel opening, but does not degrade ADPR. Channel opening also requires binding of Ca^2+^ to the transmembrane domains [38]. 8-Br-cADPR binds only to the MHR1/2 domain and stabilizes the channel at the resting state.

Cyclic ADP ribose (cADPR) can also directly activate human TRPM2, but the EC_50_ of cADPR for activation of TRPM2 is much higher than that of ADPR. This is in spite of the fact that the binding affinity of cADPR to the NUDT9-H domain is higher than that of ADPR [39]. cADPR binds to the same pocket of NUDT9-H that binds ADPR, but the interaction pattern of ADPR and cADPR with the binding sites are different [39]. It should be noted that some batches of commercially available cADPR contain 25–50% ADPR as contaminant. Activation of TRPM2 by cADPR reported in some papers may partly be due to the contaminant ADPR [40].

ADPR is the best known agonist of TRPM2, but a recent study reports that 2′-deoxy-ADPR is the most efficient endogenous agonist of the channel [41]. Compared to ADPR, 2′-deoxy-ADPR produces 10-fold more TRPM2 current. 2′-deoxy-ADPR is thought to be produced by CD38 from cytosolic 2’-deoxy-NAD. It has been speculated that 2′-deoxy-ADPR is the principal agonist of TRPM2 for mediating the physiological signaling functions [41]. ADPR-2’-phosphate is a partial agonist of TRPM2 that activates TRPM2 with reduced efficacy [40,41].

Activation of the TRPM2 by reactive oxygen species (ROS) is generally attributed to the formation of ADPR by the actions of poly(ADP-ribose) polymerase (PARP) and Poly(ADP-ribose) glycohydrolase (PARG) [42]. DNA damage activates PARP leading to the synthesis of poly (ADP-ribose) (PAR). PARG catalyzes the degradation of PARs to yield free ADPR, which activates TRPM2 leading to increase in the [Ca^2+^]_i_ and cell death [43].

*N*-(p-amylcinnamoyl) anthranilic acid, an inhibitor of TRPM2, inhibits H_2_O_2_-induced Ca^2+^ increase in the INS-1E cells [29]. Newer, more potent inhibitors of TRPM2 are curcumin, JNJ-28583113, some derivatives of 2,3-dihydroquinazolin-4(*1H*)-one, scalaradial, and 12-deacetylscalaradial [44,45,46,47]. JNJ-28583113 inhibits human TRPM2 with IC_50_ of 126 nM, scalaradial inhibits with IC_50_ of 210 nM, and the most potent derivative of 2,3-dihydroquinazolin-4(*1H*)-one inhibits with IC_50_ of 3.7 μM.

### 4.1. Role of the TRPM2 Channel in Stimulus-Secretion Coupling

The permeability ratio p_Ca_:p_Cs_ of TRPM2 is low (~0.54), but the permeability for Ca^2+^ increases (p_Ca_:p_Na_ = 5.83) when the channel is activated by heat, and activation of the channel increases [Ca^2+^]_i_ [28,29,31]. Ca^2+^ activates TRPM2 and its alternatively spliced isoforms, including the ones that do not bind ADPR [33,37]. In the presence of ADPR, TRPM2 behaves like a Ca^2+^-activated channel [33,48]. Extracellular Ca^2+^ entering through the TRPM2 channel activates the channel by binding to the Ca^2+^-binding sites located in the vicinity of the pore region. This mechanism prolongs the activation of the channel in a self-sustained manner [33,48]. Receptor-activation-induced Ca^2+^ release can also activate the TRPM2 channel [33].

TRPM2 is involved in mediating insulin secretion in response to stimulation by glucose [49,50]. Glucose-stimulated TRPM2 current and insulin secretion are reduced in rat β-cells transfected with shTRPM2 RNA indicating that TRPM2 is involved in the coupling process [51]. GLP-1-induced insulin secretion is increased in transfected β-cells where TRPM2 is overexpressed, and this increase is inhibited by 2-aminoethyl diphenyl borate. GLP-1, at nanomolar concentrations, activates the TRPM2 channel through the cAMP-Epac pathway [50,51,52]. In addition, cADPR is also involved in the activation of the TRPM2 by glucose and GLP-1 [31,53]. GLP-1 releases Ca^2+^ from the intracellular store—it is possible that this may be partly due to the release through the TRPM2, since TRPM2 channels are also present on the membrane of the Ca^2+^ stores [34,53]. TRPM2 knockout mice have higher blood glucose levels. Ca^2+^-response and insulin secretion upon stimulation by glucose and nanomolar concentrations of GLP-1 is impaired in the β-cells obtained from TRPM2 knockout mice [50]. The role of TRPM2 in stimulating insulin secretion by picomolar concentrations of GLP-1 has not been reported. Low concentration of adrenaline inhibits glucose- and GLP-1-induced insulin secretion by activating the α2A adrenoceptor, inhibiting cAMP signaling and thereby inhibiting the TRPM2 channel [54]. Nanomolar concentrations of ghrelin inhibit glucose-induced insulin secretion by inhibiting cAMP formation and thereby reducing the TRPM2 current [55].

Acidic cytoplasmic pH inhibits TRPM2 and such inhibition is abolished by high pH [56]. In this context, it is noteworthy that stimulation of β-cells by glucose increases cytoplasmic pH [57], which is supposed to favor the activation of the TRPM2 by ADPR and Ca^2+^.

### 4.2. Heat as a Regulator of TRPM2

TRPM2 is a thermosensitive TRP channel (Q_10_ = 15.6). In the presence of ADPR the Q_10_ value increases to 44. The temperature threshold and the temperature for optimal activity are ~34 °C and ~37 °C respectively. At the physiological body temperature, the TRPM2 channels are constitutively active contributing to the background depolarizing current. β-cells are rich in mitochondria, and it is known that stimulation of β-cells by glucose generates heat, which may possibly increase the local temperature and increase the activity of the TRPM2 channel [58,59]. Heat-evoked increase in [Ca^2+^]_i_ in mouse β-cells is abolished in TRPM2 knockout mice [50]. The steep temperature dependence of glucose-induced insulin secretion may partly be mediated by the temperature sensitivity of the TRPM2 channels of β-cells [60]. It is noteworthy that β-cells have other TRP channels including TRPM4, TRPM5, TRPV1, TRPV2, and TRPV4 that are temperature sensitive. Although there is much skepticism, it has been demonstrated in other systems that temperatures inside the cells can increase dramatically, but it remains unclear whether such increases in the temperature have any signaling functions [61].

### 4.3. TRPM2 and β-Cell Death

Oxidative stress increases the concentration of ADPR and Ca^2+^ in the cytoplasm, which synergize to activate TRPM2 and increase Ca^2+^ influx [29,62]. In rat insulinoma cell lines, H_2_O_2_ and TNFα cause cell death, which can be inhibited by treatment with antisense-TRPM2 [30,34,63]. Alloxan-induced β-cell death is probably mediated by Ca^2+^ influx through the TRPM2 channel [26]. Human β-cells express a short isoform of TRPM2 (TRPM2-S) [29], which does not form a channel, but inhibits the full length isoform of the channel (TRPM2-L), and by that way inhibits cell death [36,64]. This could possibly be one of the many reasons why human β-cells are relatively resistant to alloxan [65]. Production of reactive oxygen species by free fatty acids and cytokines causes β-cell death by mechanisms that involve TRPM2 [66]. It is possible that TRPM2 provides the β-cells a mechanism to undergo apoptosis when they are severely damaged by oxidative stress [67].

TRPM2 plays a role in mediating free fatty acid- and cytokine-induced β-cell death [66]. Palmitate activates NADPH-oxidase-2 and thereby generates reactive oxygen species leading to the activation of TRPM2. This leads to an increase in the concentration of Zn^2+^ by activation of the TRPM2 channels located on the lysosomes [34,68]. This, in turn, leads to an increase in the concentration of Zn^2+^ in the mitochondria, and recruitment of dynamin-related protein Drp-1 to mitochondria, which catalyzes mitochondrial fission, loss of mitochondrial membrane potential, and mitochondrial fragmentation [66]. TRPM2 knockout mice are resistant to β-cell loss and hyperglycemia caused by multiple low dose streptozotocin [68].

### 4.4. TRPM2 Channels Located on the Intracellular Membranes

In β-cells TRPM2 channels are located also on the membranes of the lysosomes. Activation of these TRPM2 channels releases Ca^2+^ from the lysosomal Ca^2+^ store [34]. Ca^2+^ released from lysosomal stores externalizes phosphatidyl serine, a distinct feature of apoptosis [69].

Activation of the lysosomal TRPM2 channel also releases Zn^2+^ from the lysosomal stores. It is possible that Zn^2+^, rather than Ca^2+^, plays a more important role in mediating apoptosis of β-cells [68]. TRPM2 mediates free fatty acid- and cytokine-induced β-cell death by releasing Zn^2+^ from the intracellular stores, and by increasing the concentration of Zn^2+^ in the mitochondria [66].

## 5. TRPM3

TRPM3 has numerous isoforms generated by alternative splicing. The TRPM3α2 isoform is more permeable for Ca^2+^ and Mg^2+^ than the TRPM3α1, which is more monovalent cation selective [70]. Extracellular monovalent cations inhibit the channel. TRPM3α2 is present in mouse β-cells and is absent in α-cells [71]. TRPM3 is present also in human β-cells but it is not known which isoforms of TRPM3 are present in these cells (Figure 1) [7].

TRPM3 channel can be activated by extracellular application of micromolar concentrations of pregnenolone sulphate, an endogenous steroid, which is also able to change activity of several other ion channels. A more potent synthetic activator of TRPM3 is 3,4-dihydro-*N*-(5-methyl-3-isoxazolyl)-a-phenyl-1(*2H*)-quinolineacetamide (CIM0216) (pEC_50_ 0.77 μM) [72]. Activation of the TRPM3 by pregnenolone or CIM0216 increases [Ca^2+^]_i_ and stimulates insulin secretion, and these effects are lost in the islets obtained from *Trpm3*^−/−^ mice [71,72,73]. TRPM3 is permeable to both Ca^2+^ and Na^+^ (p_Ca_:p_Na_ = 1.57). It is possible that pregnenolone sulphate depolarizes the β-cell first by inducing a Na^+^ current through the TRPM3 channel, and the resulting depolarization activates the voltage-gated Ca^2+^ channels. In INS-1 cells and mouse islets, mefenamic acid selectively inhibits the [Ca^2+^]_i_-increase triggered by pregnenolone sulphate, but not that triggered by glucose [73].

Pregnenolone sulphate-induced activation of TRPM3 increases the expression of the transcription factor Egr-1 which binds to the regulatory region of the transcription factor *Pdx-1* gene leading to increased insulin gene transcription [74].

TRPM3 may be involved in mediating activation of the β-cells by insulin secretagogues in several ways. TRPM3 shows constitutive activity [75], and by that way could provide the background depolarizing current necessary for membrane depolarization and electrical activity of the agonist-stimulated β-cells. Like several other channels, the TRPM3α2 channel is also positively regulated by phosphatidylinositol 4,5-biphosphate (PIP_2_) [76]. It is conceivable that the glucose-induced increase in the concentration of PIP_2_ in the plasma membrane of β-cells [77] favors activation of the channel by unidentified agonists. TRPM3 is also a thermosensitive channel [72]—it is conceivable that glucose-induced heat production [59] could promote increased activity of the TRPM3 channel.

The physiological importance of TRPM3 in human β-cells remains unclear. The channel is also highly permeable for Zn^2+^ ions and β-cells take up Zn^2+^ through this channel even when the concentration of Zn^2+^ in the extracellular solution is low [78]. In *Trpm*3^−/−^ mice, fasting blood glucose is normal and they appear generally healthy [79]. This is not surprising given that β-cells have many other TRP channels, which could possibly compensate for the loss of the TRPM3 channel in the knockout mice. It will be useful to know if these mice develop signs of pre-diabetes or diabetes when put on a high fat diet. Primidone, a medicine used in the treatment of epilepsy and essential tremor, inhibits TRPM3 with an IC_50_ of 0.6 μM [80]. This drug does not cause impaired glucose tolerance, pre-diabetes, or diabetes, suggesting that TRPM3 is not essential for insulin secretion.

## 6. TRPM4

TRPM4 is a nonselective monovalent cation channel impermeable to divalent cations [81]. It is activated by Ca^2+^, followed by a fast desensitization to activation by Ca^2+^. In CRI-G1 rat insulinoma cells, a ~25 pS TRPM4-like current activated by Ca^2+^ was first described by Sturges et al. in 1986 [82]. It was inhibited by different adenine nucleotides but the potency sequence for inhibition (AMP > ADP > ATP > adenosine) was different from that for the inhibition of the cloned TRPM4 expressed in heterologous systems (ADP > ATP > AMP >> adenosine) [83]. Leech and Habener reported a ~25 pS nonselective cation current activated by Ca^2+^ and inhibited by ATP in HIT-T15 cells [84]. This current, which is also activated by GLP-1, appears to be mediated by TRPM4.

There are at least three isoforms of TRPM4: the full length TRPM4 (TRPM4b), an N-terminal 174 amino acid deletion isoform (TRMM4a), and an isoform lacking 537 amino acids (TRPM4c) [85]. It is not known which of these are expressed in the β-cells. TRPM4 protein has two ABC transporter signature-like motifs, and four nucleotide binding domains. Consistent with these, TRPM4 is inhibited by the sulphonylurea drug glibenclamide in some cells [86]. The TRPM4-like channel of the β-cells is not inhibited by glibenclamide [87]. TRPM4 is also inhibited by cytoplasmic ATP and other adenine nucleotides without requiring Mg^2+^. While ATP inhibits TRPM4, it also inhibits the desensitization of the channel by Ca^2+^ [88]. TRPM4 has phosphorylation sites for protein kinase A (PKA), PKC, and binding sites for PIP_2_. Consistent with these, TRPM4 is regulated by PKC and PIP_2_ [88,89]. PKC phosphorylation enhances the sensitivity of the TRPM4 for activation by Ca^2+^. Different PKC isoforms regulate β-cell functions [43]. PIP_2_ moves the voltage-activation curve of TRPM4 towards negative voltages and prevents desensitization of the channel by Ca^2+^ [88]. It is noteworthy that stimulation of the β-cells by glucose increases the concentration of PIP_2_ in the plasma membrane. This is likely to promote inward depolarizing currents through the TRPM4 and activation of the voltage-gated Ca^2+^ channels [77,90].

TRPM4 is present in human β-cells [7,91], rodent islets, and a variety of rodent insulinoma cells (Table 1). It is also present in α-cells, where it plays a role in mediating glucagon secretion [92]. TRPM4 is activated by an elevated [Ca^2+^]_i_ (EC_50_ ~ 0.57–1.25 μM). Activation of the TRPM4 current by [Ca^2+^]_i_ is biphasic with a first phase that develops within seconds, and a second phase that develops slowly. The latter phase appears to be due to incorporation of TRPM2 into the plasma membrane following exocytosis [91,93]. TRPM4 is involved in mediating agonist-induced insulin secretion [91,93,94]. Suppression of the TRPM2 by a dominant negative construct inhibits the magnitude of the [Ca^2+^]_i_-increase and insulin secretion [91,93]. Inhibition of the TRPM4 by 9-Phenanthrol inhibits glucose- and GLP-1-stimulated insulin secretion from rat islets [94].

TRPM4 is involved in mediating stimulation of insulin-secretion by picomolar concentrations of GLP-1 [94,95]. Picomolar concentrations of GLP-1 activate TRPM4 and TRPM5 through activation of PKC leading to extracellular Na^+^-dependent membrane depolarization [95]. PKC-dependent phosphorylation of TRPM4 increases the sensitivity of the channel to activation by Ca^2+^ [88]. Islets obtained from the *Trpm4*^-/-^ mice respond normally to stimulation by glucose, but not to stimulation by picomolar concentrations of GLP-1 [95,96].

TRPM4 is present in the glucagon-secreting α-TC1-6 cells and INR1G9 cells [91,92]. [Ca^2+^]_i_ activates TRPM4-like Na^+^ current in the α-TC1-6 cells. Agonist-induced Ca^2+^-response and glucagon secretion is reduced in cells where TRPM4 is knocked down by TRPM4 shRNA [92].

## 7. TRPM5

TRPM5, which is closely related to TRPM4, is another nonselective cation channel activated by [Ca^2+^]_I_ [81]. Unlike TRPM4, TRPM5 is not inhibited by adenine nucleotides or glibenclamide. TRPM5 is abundant in the taste buds and is best known for its role in mediating taste signaling. It is expressed, together with the TRPM4, in rodent islets and rodent insulinoma cell lines (Table 1). In human islets, TRPM5 is almost absent in the β-cells, but it is expressed in the non-β-cells of the islet (Figure 1) [7]. However, the RNA-sequencing data were obtained from only two preparations of human β-cells and should therefore be interpreted with some caution [7].

In mice, TRPM5 is involved in mediating the glucose-induced oscillations in the membrane potential, and [Ca^2+^]_i_. In *Trpm5^−^*^/*−*^ mice the frequency of the glucose-induced fast oscillations in the membrane potential, and the fast oscillations in [Ca^2+^]_i_ are reduced. TRPM5 contributes a depolarizing current during an inter-burst interval to change the membrane potential to the threshold for starting a new burst activity. It reduces the inter-burst interval and increases the amplitude and frequency of the membrane depolarizations and action potentials [97]. Consistent with these, glucose-induced insulin secretion is reduced in the *Trpm5*^−/−^ mice, and these mice have impaired glucose tolerance [98]. In in vitro experiments, insulin secretion in response to glucose from islets isolated from *Trpm5^−^*^/*−*^ remains normal, but insulin secretion in response to GLP-1 becomes impaired [95]. Impairment of glucose-induced insulin secretion and glucose intolerance observed in in vivo experiments using *Trpm5^−^*^/*−*^ mice could partly be due to the inability of GLP-1 to trigger the downstream signals in the β-cells.

Factors that couple glucose stimulation to the activation of TRPM5 may include a glucose-induced increase in the membrane potential, [Ca^2+^]_i_, concentration of cytoplasmic arachidonic acid [99,100], and the concentration of PIP_2_ [77,101]. GLP-1-induced stimulation of insulin secretion is coupled to the activation of the TRPM5 channels by PKC [95]. TRPM5 activators improve insulin secretion from mouse islets. Steviol glycosides potentiate Ca^2+^-dependent activity of the TRPM5 channel, and by that way improve glucose-induced insulin secretion, and prevent high-fat-diet-induced hyperglycemia in mice [102].

Wolfram syndrome (diabetes insipidus, diabetes mellitus, optic atrophy, DIDMOAD) caused by mutation in the Wolframin gene (*WFS1*), is an autosomal recessive disorder. In *Wfs1^−^*^/*−*^ mice the number of islets in the pancreas is reduced, and insulin secretion from the individual islets is also reduced. In these islets the *trpm5* gene is downregulated [103].

Sweet-taste receptors are present not only in the tongue but also in the β-cells. These GPCRs are heterodimers of T1R2 (taste receptor type 1 member 2) and T1R3 (taste receptor type 1 member 3). Fructose stimulates insulin secretion by activating the sweet-taste receptors of the β-cells. TRPM5 mediates the effects of activation of the sweet-taste receptor in mouse islets [104]. *Trpm5^−^*^/*−*^ mice lack sweet-taste preference. These mice gain less weight when put on a high-calorie diet, and their glucose tolerance remains better than that of the wild type mice [105,106].

Insulin downregulates TRPM5 in the islets [107]. Hyperinsulinemia reduces mRNA expression of *Trpm5* in the islets of mouse models of obesity and diabetes (*ob/ob* and *db/db* mice) [107]. In humans, some genetic variations within the *TRPM5* locus are associated with impaired insulin secretion, increased plasma glucose concentration, reduced concentration of GLP-1, and decreased insulin sensitivity [108,109]. In the white German population, the SNP rs2301699 is significantly associated with glucose-stimulated insulin secretion in women. The minor allele carriers of the SNPs rs800344, rs800345, and rs2301699 show significantly higher glucose level during an oral glucose tolerance test and show reduced insulin sensitivity [108]. In the Turkish population, the SNP rs4929982 polymorphism is associated with metabolic syndrome. In this population an increase in the A allele and decrease in the G allele of rs4929982 polymorphism increase susceptibility to metabolic syndrome [109].

## 8. TRPM6 and TRPM7

TRPM7 (formerly called LTRPC7) is a “chanzyme” containing a serine-threonine α-kinase domain on its intracellular C-terminus. It is a nonspecific divalent cation channel that is permeable to Ca^2+^, Mg^2+^, and Zn^2+^. TRPM7 is constitutively active and by that way it provides a mechanism for background entry of divalent cations into the cells. TRPM7 is one of the most abundant TRP channels expressed in human β-cells (Figure 1) [7]. This is not surprising given that expression of TRPM7 is almost ubiquitous. In mouse islets, expression of TRPM7 is eight times higher than that of TRPM6, which is the other Mg^2+^ channel present in the mouse islets [110]. In human β-cells TRPM6 is not expressed. TRPM7 is thought to regulate intracellular Mg^2+^ concentration. Deficiency of TRPM7 reduces total cellular Mg^2+^ at least in some cells [111]. Knockdown of *Trpm7* in INS-1 cells by siRNA increases insulin secretion in response to glucose [110]. It appears that TRPM7 plays a role in mediating glucose-induced insulin secretion possibly by regulating the cytoplasmic Mg^2+^ concentration.

In some cells, TRPM7 is also present on some special intracellular vesicles (called M7 vesicles or M7V) that contain Zn^2+^ [112]. It is not known whether such vesicles exist in β-cells. If so, TRPM7 of β-cells could release Zn^2+^ from such stores in response to oxidative stress and could damage the β-cells.

TRPM7 provides a mechanism for entry of divalent trace metal ions into the cells with a permeability sequence of Zn^2+^ ≈ Ni^2+^ >> Ba^2+^ > Co^2+^ > Mg^2+^ ≥ Mn^2+^ ≥ Sr^2+^ ≥ Cd^2+^ ≥ Ca^2+^ [113]. It is conceivable that entry of toxic heavy metal ions through the TRPM7 channels of the β-cells may damage these cells, leading to the development of diabetes [114]. There is no evidence that variations in the TRPM6 or TRPM7 genes are associated with T2D [115]. TRPM6 mRNA is expressed at a low level in mouse islets but not in human β-cells [7,110].

## 9. TRPV1

Human islets and human insulinoma cells do not express TRPV1 [7,116]. TRPV1 is expressed in the rat insulinoma INS-1E cells [116,117]. In the RINm5F cells, TRPV1 can be detected at the mRNA level, but TRPV1 currents cannot be demonstrated [118]. According to most studies, primary rodent β-cells do not express TRPV1 [116,118,119,120]. TRPV1 immunoreactivity has been demonstrated in primary rat β-cells [121], but no TRPV1 current can be detected in these cells [118]. Some studies have shown that the TRPV1 channel is involved in mediating insulin secretion from isolated rodent islets or β-cells, but this is not a universal finding [118,121,122]. It is possible that capsaicin, an agonist of TRPV1, stimulates insulin secretion from these cells by some non-specific mechanisms [118,121]. Glucose-induced insulin secretion from isolated β-cells obtained from *Trpv1*^−/−^ mice is not impaired [118].

TRPV1 is also expressed in the sensory neurons that innervate the pancreas and the islets of mice and rats [119,120]. However, in adult human pancreas and islets, we cannot detect TRPV1-positive neurons by immunohistochemistry [116]. In the non-obese diabetic (NOD) mice, these neurons appear to control access of lymphocytes to the islets and play a role in the pathogenesis of autoimmune diabetes [120]. Two missense mutations in the *Trpv1* gene are associated with autoimmune diabetes in these mice. In humans, the SNP rs222747 (M315I) variant of the *TRPV1* gene is significantly increased in the type 1 diabetic patients in an Ashkenazi Jewish population, suggesting that TRPV1 may be a susceptible gene for type 1 diabetes in some ethnic groups [123].

In *Trpv1*^−/−^ mice, both normal [118] and impaired [124] glucose tolerance upon intraperitoneal injection of glucose has been reported. This difference can be due to the difference in the bodyweight of the *Trpv1*^−/−^ mice used in these studies [118,124]. TRPV1 of the sensory neurons that innervate the pancreas and the islets participate in the regulation of insulin secretion through the release of calcitonin-gene-related peptide (CGRP) and substance P [124]. In the *Trpv1*^−/−^ mice, insulin secretion in response to intraperitoneal injection of glucose is reduced, and these mice have impaired glucose tolerance [124]. It should be noted that the results obtained in *Trpv1*^−/−^ mice are different from those obtained in mice where TRPV1-positive nerve fibers are ablated by chemicals. Whole body denervation of TRPV1-positive sensory neurons by capsaicin enhances glucose-induced insulin secretion in the male mice [125,126]. CGRP and substance P can exert stimulatory or inhibitory effect on insulin secretion depending on concentrations of the peptides, glucose concentration, and animal species [124]. High concentration of CGRP inhibits insulin secretion. Ablation of the TRPV1-positive neurons increases insulin secretion by removing the inhibition.

Another mechanism by which TRPV1 increases insulin secretion involves the incretin hormone GLP-1. Activation of the TRPV1 channels in the GLP-1-secreting L-cells in the ileum stimulates GLP-1 secretion, and by that way increases insulin secretion in mice [127]. Chronic dietary capsaicin increases plasma GLP-1 and lowers plasma glucose in the diabetic *db/db* mice [127]. In animal experiments, activation of TRPV1 by pharmacological agents stimulates insulin secretion in normal mice but not in *Trpv1*^−/−^ mice by mechanisms that may involve GLP-1 or peptides released from the nerve terminals [121,122,124].

In mice, loss of TRPV1 increases obesity and insulin resistance induced by a high-fat-diet and aging [128]. *TRPV1* gene polymorphism is associated with a risk of developing T2D in humans. The minor alleles of two *TRPV1* variants rs161364 and rs8065080 are associated with reduced insulin resistance and decreased risk of T2D [129]. People with the major allele of the *TRPV1* variants rs161364 and rs8065080 have a high risk of developing T2D if their fat intake is high.

## 10. TRPV2

TRPV2 is not expressed in human β-cells but is expressed in the non-β-cells of human islets [7]. In isolated mouse β-cells and MIN6 cells, glucose-induced osmotic cell swelling activates TRPV2 leading to membrane depolarization and insulin secretion [130]. TRPV2 also displays some spontaneous activity and contributes to the background depolarizing current. In MIN6 cells, insulin accelerates exocytosis by translocation of TRPV2 to the plasma membrane, which is mediated by phosphatidylinositol 3 kinase (PI3K) [131,132]. Glucose-stimulated insulin secretion promotes translocation of TRPV2 to the plasma membrane providing a positive feedback mechanism for increased insulin secretion [132]. In MIN6 cells, the anti-aging gene *Klotho* enhances glucose-induced Ca^2+^-response and insulin secretion by translocating TRPV2 to the plasma membrane [133].

## 11. TRPV3 and TRPV4

TRPV3 and TRPV4 are absent in the human β-cells (Figure 1) [7]. TRPV4 is expressed, at low levels, in the non-β-cells of human islets [7]. TRPV4 is a thermosensitive, mechanosensitive and, osmo-sensitive channel. The difference between the mechanosensitive channels and the thermosensitive molecules lies in the size and the organization of the exciting agent [134]. The thermal stimuli represent a lot of non-coordinated events and mechanical stimuli represent net stretch. This explains why some members of the TRPV family are thermosensors, osmo-sensors and mechanosensors [134].

In MIN6 cells, TRPV4 acts as a stretch-activated ion channel. In these cells aggregated human islet amyloid polypeptide increases [Ca^2+^]_i_ by activating the mechanosensitive TRPV4 channel [135]. In INS-1E cells and rat islet cells, activation of the TRPV4 channel by thermal stimulation, hypotonic solution, or by pharmacological agonist 4α-phorbol 12,13-didecanoate (4-αPDD) increases [Ca^2+^]_i_ and stimulates insulin secretion [136]. In INS-1E cells and rat islets, short activation of TRPV4 by pharmacological agonist GSK1016790A increases insulin mRNA expression by increasing ERK1/2 phosphorylation, but prolonged activation of TRPV4 suppresses the expression of insulin mRNA, and causes death of the cells by increased production of nitric oxide [137].

## 12. TRPV5 and TRPV6

TRPV5 and TRPV6 are structurally related highly Ca^2+^-selective TRP channels present mostly in the Ca^2+^-transporting epithelial cells. TRPV5 is not present in human islets, but TRPV6 is expressed in the non-β-cells of human islets, which are mostly the α-cells [7]. It is expressed in the α-cells of mouse islets, rat β-cells, MIN6 cells, and INS-1E cells [130,138]. In INS-1E cells Ca^2+^ influx through the TRPV6 channel regulates insulin gene expression, cell viability, and cell proliferation [138].

## 13. TRPML

The three members of the transient receptor potential mucolipin (TRPML) channels are TRPML1, TRPML2, and TRPML3. TRPML1 and TRPM3 are highly expressed in human β-cells, and in other cells of human islets, but TRPML2 is not expressed in human islets (Figure 1) [7]. It is known that TRPML1 and TRPML3 are expressed almost ubiquitously, while the expression of TRPML2 is more restrictive. These channels can form hetero-multimers.

TRPML channels are located on the intracellular vesicles, especially on the late endolysosomes, but the channels are translocated to the plasma membrane in an activity-dependent manner. These channels are permeable to many cations including Na^+^, Ca^2+^, Fe^2+^, and Zn^2+^. These channels are activated by phosphatidylinositol 3,5-bisphosphate, and this phosphoinositide is enriched in the endolysosomes. Low pH in the lysosomes favors the activation of TRPML1 and high pH in the extracellular space favors the inhibition of the channel [139]. These channels play important roles in vesicular trafficking, lysosomal biogenesis, lysosomal exocytosis, and autoohagy [140]. Inactivating mutations in TRPML1 impair lysosomal functions causing accumulation of heterogenous macromolecules in the lysosomes giving rise to a severe disease called mucolipidosis type IV [141]. Some mutations of TRPML1 increase activity of the channel causing constitutive activation of lysosomal exocytosis, and increased plasma membrane localization of the channel [142]. Overactivity of the TRPML channels located on the plasma membrane can damage the cells by Ca^2+^ overload [143].

## 14. TRPP

The transient receptor potential polycystic (TRPP) family has three members: TRPP1 (product of the gene *PKD2*; previously called TRPP2), TRPP2 (product of the gene *PKD2L1*; previously called TRPP3), and TRPP3 (product of the gene *PKD2L2*; previously called TRRR5). Human β-cells and other cells of the islet express TRPP1 (Figure 1), but TRPP2 and TRPP3 are not expressed in human islets [7]. TRPP1 is a nonspecific cation channel with high permeability for Ca^2+^. It is constitutively active, and it is possible that it may contribute to the background depolarizing current for depolarization of β-cells. Mutation of the *PKD2* gene that encodes TRPP1 causes autosomal dominant polycystic kidney disease, but not diabetes or impaired glucose tolerance [144].

## 15. TRPA1

Transient receptor potential ankyrin 1 (TRPA1) is a non-selective, highly Ca^2+^ permeable cation channel. Numerous compounds of diverse structures, including many irritants, environmental toxins, natural products, endogenous reactive mediators, and pharmaceutical agents can activate this channel. Many of these compounds are thiol-reactive electrophiles that activate the channel by covalent modification of the channel. Others are non-reactive, and they activate the channel by binding without covalent modifications [145].

TRPA1 is expressed in the sensory neurons and in many other tissues. The TRPA1 channel is expressed in rodent β-cells and rodent insulinoma cells, where it mediates insulin secretion when stimulated by the agonists of the channel (Table 1) [146,147]. Activators of the TRPA1 4-hydroxy-2-nonenal, allylisothiocyanate, and 15-deoxy-Δ^12,14^-prostaglandin J2 increase [Ca^2+^]_i_ in RINm5F cells by activating the channel [32]. Cinnamaldehyde, an agonist of TRPA1, stimulates insulin secretion from rat islets [148]. Activators of the TRPA1 channel induce membrane currents, membrane depolarization, action potentials, and insulin secretion in primary rat β-cells, and all these can be blocked by selective TRPA1 inhibitors [146]. The antidiabetic sulphonylurea drug glibenclamide and its derivatives activate the TRPA1 channel by interacting with some reactive cysteines, and stimulate insulin secretion from rat islets [149,150].

In mouse β-cells and INS-1 cells, catechol estrogens activate the TRPA1 channels, increase [Ca^2+^]_i_, and stimulate insulin secretion in a glucose-dependent manner [147]. These effects are inhibited by pharmacological inhibitors of TRPA1 and siRNA. 2-hydroxyestradiol, a catechol estrogen, increases insulin secretion from human islets [147]. This is in apparent contradiction to our finding that human β-cells do not express this channel [7]. It should be noted that our mRNA expression data are based on only two preparations of purified human β-cells, and it will be more informative to perform similar analysis using β-cells obtained from a larger number of human donors.

In the islets of the GK rats (a model of T2D mellitus) the expression of the TRPA1 channels is reduced [151]. The expression of the TRPA1 channels in the islets of GK rats increases when the rats are treated by a Roux-en-Y gastric bypass surgery [151]. Gastric bypass surgery leads to an increase in the plasma concentration of bile acids, which activate the nuclear farnesoid X receptor (FXR) [151]. FXR recruits histone acetyltransferase steroid receptor coactivator-1, which promotes acetylation of histone H3 and the promotion of TRPA1 leading to increased expression of the channel [151].

Streptozotocin, a toxin used for inducing diabetes in animal models, activates TRPA1 by oxidizing the critical cysteines by peroxynitrite [152]. However, β-cell damage by streptozotocin does not require the presence of TRPA1 channels since hyperglycemia of similar magnitude develops both in wild type and *Trpa1*^−/−^ mice [152].

## 16. Conclusions

Studies of the TRP channels of the islets have increased our understanding of the mechanisms of signal transduction that leads to insulin secretion. Based on the analysis of the RNA-sequencing data obtained from human β-cells, it appears that these cells express TRPC1, TRPM4, TRPM7, TRPM2, TRPM3, TRPP1, TRPML1, and TRPML3. Some of these channels are constitutively active and contribute to the background depolarization currents. Activation of these channels increases [Ca^2+^]_i_ either directly, or through promoting membrane depolarization, which activates the voltage-gated Ca^2+^ channels. When the input resistance of the β-cells is high, small currents through the TRP channels can cause marked depolarization of the β-cell membrane-potential. TRPC1 acts as an SOCE channel. TRPM2 acts as a redox sensor that may help removal of the damaged β-cells. TRPM2, TRPM4, and TRPM5 have been implicated in mediating GLP-1-induced stimulation of insulin secretion. More studies will be needed to elucidate the mechanisms by which these channels are regulated by different intermediary metabolites, hormones, neurotransmitters, and other ligands of receptors present in the islet cells. It is important to understand whether impaired regulation and functions of these channels contribute to the pathogenesis of human diabetes.

**Table 1:** TRP channels of native and transformed islet cells.

Channel	Cell Type	Method	References
TRPC1	human β-cell	RNA sequencing	[7]
	INS-1 cells, rat β-cell, rat islet	RT-PCR, WB	[10,11,12]
	MIN6 cells, mouse islet	RT-PCR, NB	[8,9]
TRPC2	MIN6 cells	RT-PCR	[8]
TRPC3	rat β-cells	EP	[20]
	mouse β-cells	pharmacological tools	[20]
	mouse and rat islets	RT-PCR pharmacological tools, microarray	[8,20,21]
TRPC4	mouse β-cell, INS-1 cell	EP	[22,23,24]
	MIN6, βTC3, INS-1, rat β-cell	RT-PCR, NB	[8,11]
TRPC5	βTC3	RT-PCR	[8]
TRPC6	MIN6	RT-PCR	[8]
	rat islet	microarray	[21]
	INS-1E	WB	[21]
TRPM2	human β-cell	RNA sequencing	[7]
	human islet	RT-PCR, WB	[29,35]
	INS-1E	EP	[29,34,44]
	RIN-5F	EP, IF, WB, RT-PCR	[30,31,32,62]
	CRI-G1	EP, RT-PCR	[28]
	HIT-T15	IF, EP	[33]
	mouse β-cell	IF, EP	[31,34,50,54]
	rat β-cell	Ca^2+^ imaging	[31]
TRPM3	human β-cell	RNA sequencing	[7]
	INS-1, mouse islet	EP, RT-PCR, NB, WB, shRNA, KO mice	[71,72,73]
	mouse β-cell	EP	[71]
TRPM4	human β-cell	RNA sequencing, IF, EP	[7,84,91,95]
	INS-1, RINm5F, HIT-T15, MIN-6, βTC3	RT-PCR, WB, EP	[84,91,93,153]
	rat islet	pharmacological tool	[94]
	mouse islet	KO mice, EP	[95]
	αTC1-6, INR1G9	EP	[91,92]
	CRI-G1	EP	[82]
TRPM5	MIN6, INS-1, human islet	RT-PCR, RNA sequencing	[7,98,107,153]
	Mouse β-cell	RT-PCR, IF, KO, EP, Ca^2+^, insulin secretion	[95,98,102,104]
	rat islet	pharmacological tool	[154]
TRPM6	mouse islet	RT-PCR	[110]
TRPM7	human β-cell	RNA-sequencing	[7]
	INS-1	RT-PCR, SiRNA	[110]
	mouse islet	RT-PCR	[110]
TRPV1	human islet	RNA-sequencing, WB	[7,116]
	INS-1	WB, pharmacological tool, RT-PCR, EP	[116,121]
	mouse islet	KO mice	[122]
	rat islet	RT-PCR	[121]
	RINm5F	RT-PCR	[121]
TRPV2	mouse islet	RT-PCR, WB, IF	[130]
	human islet	RNA-sequencing	[7]
	MIN6	RT-PCR, WB	[131,132,133]
	mouse β-cell	IF	[132]
TRPV4	MIN6	RT-PCR, Ca^2+^ imaging	[135]
	INS-1E, rat islet	RT-PCR, WB, Ca^2+^ imaging	[136,137]
TRPV6	INS-1E	RT-PCR, WB, IF	[138]
	rat islet, rat β-cell	RT-PCR, WB, IF	[138]
	human islet	RNA sequencing	[7]
	mouse α-cells	IF	[130]
TRPML1 (MCOLN1)	human β-cell	RNA sequencing	[7]
	human islet	RNA sequencing	[7]
TRPML3 (MCOLN3)	human β-cell	RNA sequencing	[7]
	human islets	RNA sequencing	[7]
TRPP1 (PKD2)	human β-cell	RNA sequencing	[7]
	human islet	RNA sequencing	[7]
TRPA1	INS-1	pharmacological tool shRNA	[147,151]
	mouse islet	pharmacological tool	[147]
	RINm5F, rat islet	RT-PCR, WB, siRNA	[32,146]
	rat β-cell	IF, EP	[146]
	rat islet	pharmacological tool, WB, RT-PCR, EP	[150,151]

EP: electrophysiology; WB: Western blot; IF: immunofluorescence; KO: knockout.
